# The Role of Robotic Cystectomy in the Salvage and Palliative Setting: A Retrospective, Single-Center, Cohort Study

**DOI:** 10.3390/cancers16223784

**Published:** 2024-11-10

**Authors:** Aldo Brassetti, Loris Cacciatore, Flavia Proietti, Rigoberto Pallares-Méndez, Alfredo Maria Bove, Umberto Anceschi, Riccardo Mastroianni, Leonardo Misuraca, Gabriele Tuderti, Giuseppe Chiacchio, Mariaconsiglia Ferriero, Rocco Simone Flammia, Costantino Leonardo, Giuseppe Simone

**Affiliations:** Department of Urology, IRCCS “Regina Elena” National Cancer Institute, 00144 Rome, Italy; aldo.brassetti@gmail.com (A.B.); flavia.proietti@ifo.it (F.P.); rigo_pallares@hotmail.com (R.P.-M.); alfredo.bove@ifo.it (A.M.B.); umberto.anceschi@gmail.com (U.A.); riccardo.mastroianni@ifo.it (R.M.); leonardo.misuraca@gmail.com (L.M.); gabriele.tuderti@gmail.com (G.T.); gipeppo1@gmail.com (G.C.); marilia.ferriero@gmail.com (M.F.); roccosimone92@gmail.com (R.S.F.); costantino.leonardo@ifo.it (C.L.); puldet@gmail.com (G.S.)

**Keywords:** bladder cancer, radical cystectomy, robot-assisted radical cystectomy, cutaneous ureterostomy, frail patients, overall survival

## Abstract

**Introduction:** This article compares surgical and survival outcomes of robot-assisted and open radical cystectomy with cutaneous ureterostomy for the treatment of frail bladder cancer patients with limited life expectancy. **Methods:** The institutional database was searched for cystectomy cases with cutaneous ureterostomy, from 1 June 2016 to 31 August 2022. The study population was split into two groups, according to the surgical approach. The baseline characteristics and surgical outcomes were compared. Logistic regression analyses identified predictors of major bleeding events (hemoglobin loss ≥ 3.5 g/dL or blood transfusion) and re-operation within 30 days from surgery. The Kaplan–Meier method estimated the impact of the robotic approach on overall survival and Cox regression analysis assessed its predictors. **Results:** A total of 145 patients were included: 30% (n = 43) underwent robotic cystectomy. Patients’ characteristics and tumor stages distribution were comparable in the two groups but those receiving a minimally invasive treatment showed significantly reduced times to flatus, bowel and hospital discharge (all *p* < 0.001). Although operation times were longer in this cohort, major bleeding events (60% vs. 89%) and postoperative severe complications (0 vs. 8%) (both *p* < 0.001) were less frequent compared to the open approach. A logistic regression showed that robotic surgery independently predicted major bleeding events (OR: 0.26; 95%CI 0.09–0.72; *p* = 0.02) but not the need for re-intervention. A Kaplan–Meier analysis showed that robotic cystectomy was associated with a significant advantage in terms of overall survival (LogRank = 0.03), and this result was confirmed by Cox regression analysis (HR: 0.39; 95%CI 0.14–0.94; *p* = 0.04). **Conclusions:** Robotic cystectomy with cutaneous ureterostomy may represent a viable option to treat frail bladder cancer patients, as the minimally invasive approach reduces the risk of bleeding and serious complications and provides a prompt restoration of bowel function and a shorter hospital stay compared to open surgery.

## Simple Summary

This paper compares robotic and open radical cystectomy with cutaneous ureterostomy in frail bladder cancer patients. Of 145 patients, 30% underwent robotic surgery, which was associated with faster recovery, fewer bleeding events, and fewer complications compared to open surgery. Robotic surgery also showed a survival benefit. The findings suggest that robotic cystectomy is a viable option for frail patients, offering quicker recovery and reduced risks.

## 1. Introduction

Bladder cancer (BCa) is a prevalent neoplasm worldwide [1]. In males, it ranks as the fourth most common tumor, accounting for 61,700 new cases per year (6% of all cancers) [2]. At the time of the initial diagnosis, 26% of patients present with a muscle-invasive disease, while regional or distant metastases are present in a quarter of cases [3]. Due to the high cancer-specific mortality (CSM), the prevalence of non-organ-confined diseases (NOCD) is lower compared to non-muscle-invasive ones [2,4]. The mean age at diagnosis is 73 years [3]. In males, the age-specific incidence exhibits a significant increase, starting from 1/100,000 per year in patients under the age of 45, rising to 25/100,000 per year in those aged between 45 and 64, and growing at a slower rate in subsequent decades [5].

Radical cystectomy (RC) is considered the treatment of choice for patients with non-metastatic muscle-invasive bladder cancer (MIBC) [6], and it is also recommended for symptomatic patients with non-organ-confined disease (NOCD) with palliative intent. However, this surgery is associated with non-negligible morbidity, particularly in elderly and frail individuals [7]. The incidence of postoperative complications ranges between 30% and 60%, with a 30-day mortality rate of 5% [8], closely related to patient age and overall health [9].

In the 1990s, minimally invasive approaches were proposed to minimize the morbidity associated with open RC (ORC) [10]. Subsequently, robotic surgery was introduced to address concerns related to the complexity of the laparoscopic approach [11,12]. Recent randomized controlled trials (RCTs) have not only demonstrated the superiority of robot-assisted RC (RARC) over ORC in terms of bleeding [13] but have also established its non-inferiority regarding postoperative complications and oncological outcomes [13,14,15,16,17].

In recent years, life expectancy has experienced a significant increase in Western countries. In 2016, 5.4% of the European population was aged over eighty [18]. Consequently, urologists are more frequently faced with the challenge of treating elderly and vulnerable individuals suffering from MIBC [19,20]. In such cases, the benefits of cystectomy must be carefully weighed against the risks associated with the treatment [21]. In this context, recent randomized clinical trials comparing RARC and ORC do not offer specific information, as frail BCa patients with limited life expectancy have been systematically excluded from enrollment [13,14,15,16,17].

In the absence of specific available evidence, our study aims to assess and compare the outcomes of open and robotic RC in fragile patients with limited life expectancy.

## 2. Materials and Methods

After receiving approval from the institutional review board (M.BBIRE.02), we conducted a retrospective analysis of our prospectively maintained database on BCa. We extracted data related to a consecutive series of patients who underwent RC with cutaneous ureterostomy (CU) at our center from 1 June 2016 to 31 August 2022. All patients with incomplete data were excluded from the analysis.

The following data were extracted:

- Patients’ demographic, anthropometric and clinical characteristics at baseline (age, gender, body mass index [BMI], American Society of Anesthesiologists [ASA] score, main comorbidities and Charlson Comorbidity Index [CCI, with patients scoring ≥ 5 defined as “severely comorbid”] [22]).
- Surgical technique and outcomes (postoperative hemoglobin drop, operation time [OT], length of hospital stay [LOS], time to flatus and complete canalization, postoperative complications [stratified according to the Clavien–Dindo scale, with those grade ≥ III considered as “severe”] [23]). Major bleeding events (MBEs) were defined as cases that exhibited either a postoperative reduction in hemoglobin of ≥3.5 g/dL or necessitated blood transfusion [24].
- Final histology and pathological stage (defined according to the American Joint Committee on Cancer [AJCC] guidelines [25]).

RC with pelvic lymph node dissection (PLND) was recommended, with curative intent, to patients with high-risk non-muscle-invasive bladder cancer (NMIBC) and those with non-metastatic muscle-invasive bladder cancer (MIBC). Additionally, it was also offered to select symptomatic patients with NOCD (cT ≥ 3 and/or cN+ and/or cM+) for palliation [6]. The decision to omit pelvic lymphadenectomy was left to the discretion of the operating surgeon, who evaluated the risks and benefits on a case-by-case basis, considering each patient’s comorbidities and the stage of disease.

To avoid the well-documented morbidity associated with bowel-based urinary diversions [26,27], cutaneous ureterostomy was routinely recommended to elderly individuals (>75 years) [28], frail patients (ASA score > 3) and those with limited life expectancy based on comorbidities (Charlson Comorbidity Index > 5) and clinical tumor stage (cT2> and/or cN+ and/or M+) [29]. Until May 31, 2021, all these procedures were performed with an open approach. Thereafter, cystectomies have been exclusively carried out using the daVinci Xi^®^ robotic platform (Intuitive Surgical, Sunnyvale, CA, USA), with a standard 3-arm configuration [30]. Detailed surgical techniques of both approaches have been previously described [26,31].

Since January 2016, our department has implemented international Early Recovery After Surgery (ERAS) protocols [32]. In the preoperative phase, we no longer perform bowel preparation, allowing patients to consume clear liquids up until 6 h before their operation. We have adopted standard anesthetic schemes during surgery to ensure euvolemia, normothermia and maintain a urinary volume of 0.5 mL/kg/h, which serves as a measure of adequate organ perfusion. Antibiotic prophylaxis is now administered only 20 min prior to skin incision. The nasogastric tube is promptly removed right after surgery, and crystalloids are administered at a fusion rate of 150 mL/h. Opioid analgesics are used sparingly, only as needed, rather than routinely prescribed. Starting from the first day after the surgery, patients are encouraged to start walking, and thromboprophylaxis with low molecular weight heparin is initiated. Simultaneously, oral hydration with clear fluids is permitted, and we encourage the gradual return to full enteral nutrition as long as there are no symptoms of nausea, vomiting or abdominal pain.

After RC, all patients underwent monthly urological outpatient visits for the replacement of ureteral stents. Those diagnosed with NOCD at final pathology were offered immuno/chemotherapy and underwent periodic evaluations with CT scans of the thorax and abdomen every 6 months for up to five years postoperatively [6].

The primary aim of our study was to compare surgical outcomes in frail BCa patients with limited life expectancy undergoing either open or robotic cystectomy with CU. Secondarily, we assessed the impact of the two surgical approaches on overall survival (OS).

### Statistical Methods

The study population was split into two groups based on surgical approach (ORC vs. RARC). Frequencies and proportions were used to report categorical variables, which were compared by means of the χ^2^-test. Continuous variables were presented as median and interquartile ranges (IQRs) and were compared using either the Mann–Whitney U test or the Kruskal–Wallis one-way test based on their normal or non-normal distribution, respectively (normality of the distribution of variables was tested by the Kolmogorov–Smirnov test). The Kaplan–Meier (KM) method was used to specifically assess the impact of the surgical approaches on OS, and the LogRank test was applied to assess statistical significance between the two groups. Binary logistic regression models were used to identify potential predictors of MBEs and re-intervention within 30 days of RC, while Cox regressions were used to investigate predictors of all-cause mortality (ACM). Statistical significance was set at *p* < 0.05 for all tests. The analysis was performed using the Statistical Package for Social Science v. 25.0 (SPSS Inc., Chicago, IL, USA).

## 3. Results

The analysis included a total of 145 patients, with a median age of 77 years (IQR: 69–80). Most of these were men (75%), with an ASA score of ≥3 (68%). Overall, 30% (n = 43) underwent RARC. Patients’ preoperative characteristics were comparable in the two groups (all *p* > 0.06), and so was the disease stage distribution at final pathology (*p* = 0.07) (Table 1). Overall, 97 patients (60%) underwent PLND, evenly distributed between the open (58%) and robotic (65%) groups (*p* = 0.36).

Operation times were significantly longer in the robotic cohort compared to the open cohort (165 min vs. 120 min; *p* < 0.001). On the contrary, recovery was faster in the former group, with times to flatus (2 days vs. 3 days; *p* < 0.001) and bowel (3 days vs. 5 days; *p* < 0.001) and hospital stay (4 days vs. 7 days; *p* < 0.001) being significantly shorter after RARC (Table 1).

Overall, 85% of patients experienced mild complications, with a significantly higher incidence in the ORC group compared to the RARC group (94% vs. 63%; *p* = 0.04) (Table 1). Among these, the most common complication was anemia requiring transfusion, accounting for 99 cases (78 vs. 21; *p* < 0.001). Additionally, 13 patients developed a fever that required both antipyretics and antibiotic therapy (11 vs. 1; *p* = 0.04). A total of nine patients experienced postoperative bowel-related complications, with seven in the open cohort and two in the robotic cohort (*p* = 0.49). Five patients in the ORC group (5%) and two in the RARC group (4%) (*p* = 0.11) had a delayed time to flatus after surgery, necessitating prolonged hospitalization and prokinetic treatment. One patient experienced profuse diarrhea after robotic cystectomy, requiring fluid therapy and probiotic supplementation. Finally, one case of bowel perforation was observed after open surgery, necessitating exploratory laparotomy and ileal resection.

**Figure 1:**
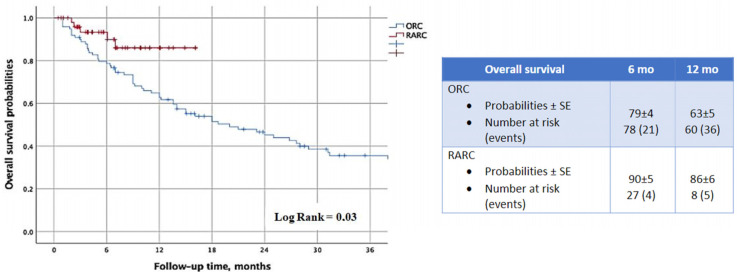
Impact of robotics approach on overall survival probabilities.

The KM analysis revealed that RARC was associated with a significant advantage in terms of OS (LogRank = 0.03) (Figure 1). This finding was further confirmed by the Cox regression model (HR: 0.39; 95%CI 0.14–0.94; *p* = 0.04) (Table 2).

Logistic regression analyses showed that the robotic approach was an independent predictor of MBEs (OR: 0.26; 95%CI 0.09–0.72; *p* = 0.02), but not of the risk of re-intervention at 1 month after surgery (Table 3).

## 4. Discussion

Despite the widespread use of robotic platforms in urology, open surgery still remains the approach of choice for RC, even in Western countries [33]. In fact, robotics has been shown to provide significant advantages in the treatment of several neoplasms by reducing morbidity without compromising oncologic outcomes [13]. Regarding its application to cystectomy, recent randomized controlled trials have demonstrated a lower incidence of intra/postoperative complications (especially related to the risk of bleeding), while ensuring non-inferior survival rates compared to those observed after ORC [15,16,17,34].

The groundbreaking CORAL study aimed to compare postoperative complication rates after ORC, RARC, and laparoscopic radical cystectomy (LRC) [17]. Although the difference between robotic and open surgery was not statistically significant (55% vs. 70%; *p* = 0.5), an important discrepancy in the 30-day complication rate emerged among the three approaches (70% vs. 55% vs. 26%; *p* = 0.02). Conversely, no significant difference was observed in the 90-day complications (70% vs. 55% vs. 32%; *p* = 0.07). The study also highlighted significant discrepancies in OT (293 min vs. 389 min vs. 301 min; *p* < 0.001), with open surgery proving significantly faster than robotic surgery (*p* < 0.001). In 2015, Bochner et al. led a RCT comparing the incidence of complications in individuals undergoing ORC vs. RARC at 90 days after surgery [15]. No statistically significant difference was observed between the two groups (66% vs. 62%; *p* = 0.7), suggesting that the minimally invasive approach did not offer any additional benefits. However, the data revealed that RARC had a longer surgical duration compared to ORC (464 min vs. 330 min; *p* < 0.001) and demonstrated superiority in terms of blood loss (516 mL vs. 676 mL; *p* = 0.027). Nix et al. [35] also reported similar evidence, confirming lower intraoperative bleeding (564 mL vs. 273 mL; *p* = 0.0003) and longer OT (3.5 h vs. 4.2 h; *p* < 0.0001) associated with the robot-assisted treatment. The RAZOR trial further supported the advantages of RARC, confirming its superiority in terms of estimated blood loss (300 mL vs. 700 mL; *p* < 0.0001) and transfusion rate (24% vs. 45%; *p* = 0.0002), while no difference was observed concerning the LOS (6 days vs. 7 days; *p* = 0.021) [16]. Interestingly, no significant differences were found in terms of oncologic outcomes, with a 2-year progression-free survival (PFS) rate of 71.6% after ORC and 72.3% after RARC (*p* = 0.90). The rate of positive surgical margins was also comparable in the two groups (5% vs. 6%; *p* = 0.59). The analysis of quality of life (QoL) endpoints using the Functional Assessment of Cancer Therapy–Vanderbilt Cystectomy index (FACT-VCI) did not reveal any significant differences between open and robotic approaches [16]. More recently, in May 2022, the first RCT comparing surgical outcomes after ORC and RARC with intracorporeal urinary diversion was published. The data from this study confirmed that the minimally invasive approach provides a significant advantage in terms of transfusion rates (22% vs. 41%; *p* = 0.046), while complications, the LOS, and QoL at 6 months post-operation were comparable between the two study cohorts [13].

However, the generalizability of these study conclusions is limited, as frail patients with a limited life expectancy and those with non-organ-confined disease were not enrolled in any case. According to our study, though, the robotic approach is confirmed to be superior to open surgery even in this specific setting. In fact, 117 MBEs were observed overall, with a significant advantage of RARC compared to ORC (60% vs. 89%; *p* < 0.001). Accordingly, a logistic regression analysis highlighted that the minimally invasive approach was associated with a significant reduction in the risk of this specific complication (OR: 0.26; 95%CI 0.09–0.72; *p* = 0.02).

Regarding the aging population, it is projected that by 2060, there will be over 100 million individuals over the age of 70 in the United States [36]. As a result, considering the current onset age of BCa [37], urologists will increasingly face the challenge of treating elderly and frail patients who have a poor performance status. This will require a careful evaluation of the risks and benefits associated with the treatments. It is believed that the fear of RC-specific morbidity and the presumed limited life expectancy of elderly patients are reasons for the undertreatment observed in octogenarians [21,38,39,40,41]. A recent analysis of the US National Cancer Database, which included 28,691 patients with MIBC treated from 2004 to 2008, revealed a significant decrease in the recommendation for cystectomy as patients’ age increased (odds ratio of 0.34 in octogenarians compared to those in their fifties; *p* < 0.001). Only 40% of individuals in the 80–89 age group underwent surgery [42]. Another analysis from the Surveillance, Epidemiology, and End Results registry highlighted that octogenarian patients with MIBC who underwent RC had a higher cancer-specific mortality rate compared to younger patients, ranging from 31.7% to 65.5% at 5 years post-operation, depending on the disease stage [43]. Furthermore, it was found that elderly patients with infiltrating carcinoma who did not receive proper treatment had a high chance of dying from cancer rather than from other age-related causes [44]. As several studies have demonstrated the feasibility of RC in the elderly [45,46,47], it is possible to conclude that chronological age should not be a limiting factor for surgical indication.

Undoubtedly, most complications observed after cystectomy can be attributed to the type of urinary diversion chosen, with the ileal conduit being the most common option. During the reconstructive phase, the manipulation and disconnection of loops from the intestinal continuity expose patients to a significant risk of canalization delays, anastomotic dehiscence and peritonitis [23]. Conversely, the CU is a simple and quick procedure that does not carry the usual risks associated with intestinal diversions. Various studies have compared surgical, functional outcomes and quality of life in frail patients who underwent non-continent UD cystectomy [45,46,48]. Deliveliotis et al. reported shorter surgical times (131 vs. 251 min; *p* < 0.001), less blood loss (387 vs. 490 mL; *p* < 0.001), limited use of transfusions (24% vs. 56%; *p* = 0.025), and a lower rate of postoperative complications (13.7% vs. 40%; *p* = 0.035) in patients undergoing CU [46]. Longo et al. also reported similar results and emphasized that quality of life was not significantly different between the two groups [49]. In our study, we observed significantly longer times to flatus (3 days vs. 2 days; *p* < 0.001) and bowel (5 days vs. 3 days; *p* < 0.001) and a longer hospital stay (7 days vs. 4 days; *p* < 0.001) in the ORC group. Moreover, severe complications (8% vs. 0%; *p* < 0.001) and the need for re-intervention (22% vs. 5%; *p* = 0.01) were more frequent in this cohort. A univariable analysis revealed a significant association between the robotic approach and the risk of re-intervention at 30 days (OR: 0.11; 95%CI 0.01–0.86; *p* = 0.04), but this finding was not confirmed at the multivariable method. According to our data, RARC also appears to be associated with an overall survival advantage, as demonstrated by the Kaplan–Meier (LogRank = 0.03) and Cox regression (HR: 0.39; 95%CI 0.14–0.94; *p* = 0.04) analyses. This can be ascribed, at least partially, to the influence of bleeding and transfusions on the health of patients undergoing open cystectomy. In fact, all recent randomized clinical trials consistently highlighted the superiority of the robotic approach in terms of reduced morbidity when compared to the open approach. However, we cannot ignore the potential impact of various lifestyle-related factors, which we failed to collect.

Our study is not devoid of limitations that need to be acknowledged. The first lies in its retrospective nature, which significantly restrains the soundness of our conclusions. The study population is limited by our institutional caseload, and the lack of a prospective design prevented us from achieving a homogeneous distribution of cases across the two cohorts. Obviously, statistical power was not computed for this retrospective study, further diminishing the significance of our observations. A further limitation of this study is the short median follow-up duration. Given the advanced age and multiple vulnerabilities of these patients, arranging regular outpatient follow-ups presents considerable challenges. Additionally, cancer-specific mortality likely contributed to the reduced median follow-up time in this population; it is worth noting that 74% of enrolled patients had a pathologic diagnosis of locally advanced or metastatic bladder cancer, which is associated with very limited survival. Another major limitation of the present study is the lack of randomization, which makes it susceptible to selection biases. Nevertheless, our aim was to report and compare surgical and oncological outcomes of frail patients with limited life expectancy undergoing radical cystectomy with cutaneous ureterostomy at our center, in different time periods and with different surgical approaches. Although the number of patients included in the analysis was small, ours is the largest series ever reported for this specific setting. Interestingly, the results of our analysis align with the findings of recent randomized clinical trials, which have demonstrated the superiority of RARC compared to ORC in terms of reduced morbidity, which may explain the survival advantage that we observed.

Another limitation stems from the fact that the data presented in this study are derived from a single-center series. In fact, our institution serves as a high-volume referral center, performing over 150 cystectomies annually (which exceeds the minimum threshold recommended by the International Consultation on BCa by a factor of 15) [47]; therefore, the results we have reported may not be reproducible in centers with a lower case load.

It is important to note that the study cohort comprise vulnerable patients with a limited life expectancy and advanced disease, who underwent cystectomy with continent urinary diversion; consequently, the outcomes herein described cannot be extended to different populations. Finally, we could not collect data concerning every possible lifestyle factor of the patients included in the analysis. However, it has been shown that marital status significantly affects life expectancy of elderly [50], along with daily physical activity [51] and dietary habits [52].

## 5. Conclusions

In frail patients with advanced stage BCa and limited life expectancy, the expected benefits of treatment must be weighed against potential complications. In this context, based on the findings of our analysis, RARC with CU may represent a viable option, as the minimally invasive approach, when compared to open surgery, appears to be associated with a reduced risk of bleeding and serious complications and seems to ensure a prompt restoration of bowel function and a shorter hospital stay.

## Figures and Tables

**Table 1:** Patient characteristics and perioperative outcomes, based on the surgical approach.

	Overall (n = 145)	ORC (n = 102)	RARC (n = 43)	*p*
Age, y	77 (69/80)	77 (69/80)	76 (71/79)	0.58
Male gender, n (%)	109 (75%)	72 (71%)	37 (86%)	0.06
BMI	25.6 (24/27.68)	25.6 (24/27.7)	25.7 (23.7/27.9)	0.97
Diabetes, n (%)	35 (24%)	24 (23%)	11 (26%)	0.79
Hypertension, n (%)	95 (65%)	68 (67%)	27 (63%)	0.65
AMI, n (%)	15 (10%)	8 (8%)	7 (16%)	0.13
ASA ≥ 3, n (%)	98 (68%)	70 (69%)	28 (65%)	0.68
CCI	4 (4/6)	4 (4/6)	4 (4/6)	0.67
NAC, n (%)	24 (17%)	17 (17%)	7 (16%)	0.95
Hb at baseline, g/dL	11.8 (10.2/13.1)	11.5 (10/12.9)	12.6 (10.7/13.4)	0.05
Hb at discharge, g/dL	9.8 (9.1/10.8)	9.5 (9/10.3)	11.1 (10/12.1)	**<0.001**
OT, min	140 (101/172)	120 (94/153)	165 (150/210)	**<0.001**
Time to flatus, d	2 (2/3)	3 (2/4)	2 (1/2)	**<0.001**
Time to bowel, d	4 (3/5)	5 (4/6)	3 (3/4)	**<0.001**
MBEs, n (%)	117 (81%)	91 (89%)	26 (60%)	**<0.001**
Postoperative compications, n (%)				
Clavien–Dindo grade < 3	123 (85%)	96 (94%)	27 (63%)	0.04
Clavien–Dindo grade ≥ 3	9 (6%)	9 (8%)	0 (0%)	**<0.001**
LOS, d	6 (4/8)	7 (5/9)	4 (3/5)	**<0.001**
AJCC stage, n (%)				0.07
0is-II	38 (26%)	22 (22%)	16 (37%)	
IIIa	80 (55%)	60 (59%)	20 (45%)	
IIIb	19 (13%)	16 (16%)	3 (7%)	
IV	8 (5%)	4 (4%)	4 (9%)	
Histopathology report, n (%)				0.77
TCC	132 (91%)	92 (90%)	40 (93%)	
SCC	6 (4%)	5 (5%)	6 (4%)	
Others	7 (5%)	5 (5%)	7 (5%)	
30d Complications, n (%)	57 (39%)	40 (39%)	17 (39%)	1.00
Severe	24 (16%)	22 (22%)	2 (5%)	**0.01**
Follow-up time, m	10 (4/23)	15 (7/32)	6 (3/10)	**<0.001**

Data reported as median (IQR), ORC = open radical cystectomy, RARC = robotic assisted radical cystectomy, AMI = acute myocardial infarction, ASA = American Society of Anesthesiologists, CCI = Charlson Comorbidity Index, NAC = neoadjuvant chemotherapy, Hb = hemoglobin, OT = operation time, MBEs = major bleeding events, LOS = length of hospital stay, AJCC = American Joint Committee on Cancer, TCC = transitional cell carcinoma, SCC = squamous cell carcinoma, 30d Complications = complications at 30-day follow-up.

**Table 2:** Cox regression to identify predictors of all-cause mortality (ACM).

	ACM							
	Univariable Analysis				Multivariable Analysis			
	HR	95% CI		*p*	HR	95% CI		*p*
		Lower	Higher			Lower	Higher	
Age	0.98	0.95	1.02	0.38	-	-	-	-
Male gender	0.78	0.46	1.32	0.36	-	-	-	-
BMI	0.97	0.90	1.03	0.34	-	-	-	-
ASA ≥ 3	1.18	0.69	2.01	0.54	-	-	-	-
CCI	1.07	0.90	1.27	0.41	-	-	-	-
Robotics approach	0.39	0.14	0.94	0.04	-	-	-	-
OT	1.01	0.99	1.01	0.84	-	-	-	-

ACM = all-cause mortality, BMI = body mass index, ASA = American Society of Anesthesiologists, CCI = Charlson Comorbidity Index, OT = operative time.

**Table 3:** Logistic regression to identify predictors of major bleeding events (MBEs) and the need for re-intervention 30 days after cystectomy.

	MBEs								30 d Re-Intervention							
	Univariable Analysis				Multivariable Analysis				Univariable Analysis				Multivariable Analysis			
	OR	95% CI		*p*	OR	95% CI		*p*	OR	95% CI		*p*	OR	95% CI		*p*
		Lower	Higher			Lower	Higher			Lower	Higher			Lower	Higher	
Age	0.98	0.92	1.04	0.49	-	-	-	-	1.02	0.95	1.09	0.59	-	-	-	-
Male gender	0.19	0.04	0.83	0.03	0.21	0.04	1.01	0.05	5.66	0.71	45.1	0.10	-	-	-	-
BMI	1.02	0.89	1.15	0.79	-	-	-	-	1.04	0.89	1.22	0.63	-	-	-	-
ASA ≥ 3	1.20	0.50	2.85	0.68	-	-	-	-	2.67	0.71	9.99	0.14	-	-	-	-
CCI	0.88	0.66	1.15	0.35	-	-	-	-	1.31	0.92	1.86	0.13	-	-	-	-
Robotics approach	0.18	0.08	0.44	<0.001	0.26	0.09	0.72	0.01	0.11	0.01	0.86	0.04	0.33	0.04	3.07	0.33
OT	0.99	0.98	0.99	0.02	0.99	0.98	1.00	0.39	0.97	0.98	0.99	0.01	0.98	0.96	0.99	0.02

MBEs = major bleeding episodes, 30 d re-intervention = re-intervention 30 days after cystectomy, BMI = body mass index, ASA = American Society of Anesthesiologists, CCI = Charlson Comorbidity Index, OT = operative time.

## Data Availability

The data presented in this study are available on request from the corresponding author.

## Abbreviations

Radical cystectomy	RC
open radical cystectomy	ORC
robot-assisted radical cystectomy	RARC
cutaneous ureterostomy	CU
bladder cancer	BCa
cancer-specific mortality	CSM
non-muscle-invasive bladder cancer	NMIBC
muscle-invasive bladder cancer	MIBC
neoadjuvant chemotherapy	NAC
randomized control trials	RCTs
Body Mass Index	BMI
American Society of Anesthesiologists score	ASA
length of stay	LOS
Clavien–Dindo scale	CD scale
major bleeding events	MBEs
non-organ-confined disease	NOCD
Early Recovery After Surgery	ERAS
all-cause mortality	ACM
overall survival	OS
laparoscopic radical cystectomy	LRC
quality of life	QoL
